# Factors associated with psychological distress among family caregivers of preschool children with autism: an analysis

**DOI:** 10.3389/fpsyt.2026.1570322

**Published:** 2026-05-20

**Authors:** Song Chen, Yuqi Tian, Ruixin Ma, Fashui Gao, Guofang Ma

**Affiliations:** 1 School of Health Management, Xinjiang Medical University, Urumqi, Xinjiang, China; 2 School of Public Health, Xinjiang Medical University, Urumqi, Xinjiang, China; 3 Institute of Health Management, Xinjiang Medical University, Urumqi, Xinjiang, China

**Keywords:** family caregivers, LASSO regression, preschool children with autism, psychological distress, random forest algorithm

## Abstract

**Objective:**

To identify factors associated with psychological distress among family caregivers of preschool-aged children with Autism Spectrum Disorder (ASD) using LASSO regression and random forest algorithms.

**Methods:**

A convenience sampling method was employed to recruit 213 caregivers of preschool-aged children with ASD from three institutions in Urumqi between December 2023 and October 2024. Participants completed a demographic questionnaire and the Symptom Checklist-90. Predictors were screened through LASSO regression, and a random forest risk assessment model was constructed and validated on the test set. A logistic regression model was simultaneously developed for comparative validation.

**Results:**

The top five factors associated with caregivers’ psychological distress are comorbid conditions in children with ASD, daily care hours, marital status, the severity of the child’s ASD, and employment status. The model outperformed logistic regression on both the training set (AUC = 0.845, sensitivity=0.893, specificity=0.913, accuracy=0.933, F1 score=0.901) and test set (AUC = 0.87, sensitivity=0.733, specificity=0.727, accuracy=0.710, F1 score=0.721). Decision curve analysis demonstrated clinical utility across threshold probability ranges of 0–0.85.

**Conclusion:**

Factors associated with psychological distress among autism caregivers include comorbidity status, caregiving duration, marital status, disease severity, and employment status. These findings provide evidence-based guidance for early psychological intervention targeting high-risk caregivers.

## 1 Introduction

Autism Spectrum Disorder (ASD) is a neurodevelopmental disorder characterized by deficits in social communication and interaction, as well as repetitive, compulsive behaviors and restricted interests and activities (1). Currently, ASD is one of the most commonly diagnosed disorders in children. According to the “China Autism Education Rehabilitation Industry Development Status Report” released in 2015, the prevalence of ASD has been rising steadily, from 1 in 88 in 2009 to 1 in 54. This increasing prevalence has led to a growing number of caregivers, who subsequently experience significant physical and psychological burdens. ASD in preschool children profoundly impacts family functioning, parent-child relationships, school readiness, self-awareness, emotions, and social behaviors—all of which are crucial for establishing a healthy emotional and psychological foundation (2).

Numerous studies have shown that parents of children with ASD often experience elevated levels of parenting stress, depression, and anxiety (3, 4). Caregivers, who are typically the primary source of social and emotional support for preschool children with ASD, play an essential role during this developmental stage. Moreover, as preschool children are still developing cognitive, emotional, and social abilities, they lack self-care skills and rely heavily on parents or grandparents for daily care (5). The long-term management of ASD requires intensive, prolonged intervention, which further exacerbates the burden on caregivers. Long-term caregiving may lead to psychological issues such as anxiety and depression, which not only harm the caregivers’ own physical and mental health but also compromise the quality of care they provide, thereby influencing treatment outcomes and rehabilitation effects for the children (6).

Previous research has shown that elevated levels of caregiver strain—a subjective experience of stress and burden—and clinically significant depressive symptoms are each associated with reduced parenting competence. This, in turn, may negatively affect the child’s physical and emotional health, potentially creating a detrimental cycle (7–9). Furthermore, the lifelong need for support in areas such as education, health care, and community services—depending on disease severity—makes ASD a major societal challenge (10). These challenges directly impact key Quality of Life domains: material well-being through financial strains, social inclusion through limited participation in community activities, and personal development through constrained opportunities for growth and self-actualization.

Given the complex and multifactorial nature of caregiver burden and its theoretical underpinnings in QOL and stress-coping frameworks (11, 12), traditional analytical approaches may be insufficient to fully elucidate the underlying risk factors. Based on these challenges, recent advances in machine learning (ML) provide new tools for identifying complex patterns in high-dimensional data. ML is a computational method aimed at uncovering underlying relationships between variables in complex datasets, iteratively improving model accuracy and efficiency through data input and model output. It has been successfully applied to early detection, real-time prediction, auxiliary diagnosis, and prognosis of various diseases (13, 14). As a non-parametric ensemble machine learning method, the Random Forest algorithm is particularly effective at identifying optimal variable combinations for predicting new observations and has significant advantages in predicting mental health outcomes (15, 16).

This study aims to identify factors influencing psychological distress among caregivers of preschool children with ASD using the least absolute shrinkage and selection operator (LASSO) algorithm. The Random Forest (RF) method is applied to rank feature importance and establish a risk assessment model for psychological distress in this caregiver population. This approach not only identified specific factors affecting the development of psychological distress but also provides etiological clues for related research and theoretical support for early mental health interventions for caregivers of preschool children with ASD.

## 2 Data and methods

### 2.1 Study object

Based on the nature and developmental positioning of the institutions, a convenience sampling method was adopted. The primary caregivers for the rehabilitation of children with Autism Spectrum Disorder were selected from three institutions: a tertiary grade A children’s hospital, a specialized tertiary hospital, and a private children’s rehabilitation institution, during the period from December 2023 to October 2024.

#### 2.1.1 Inclusion criteria

(1) Children aged 3–6 years with Autism Spectrum Disorder, as defined by the 5th edition of the Diagnostic and Statistical Manual of Mental Disorders. (2) Caregivers are over 18 years old and have the ability to care for the children. (3) No fees are charged for caregiving. (4) Caregivers have good communication, cognitive, and understanding abilities.

#### 2.1.2 Exclusion criteria

(1) Caregivers suffering from psychological or psychiatric diseases. (2) Caregivers who had received psychological counseling or psychotherapy for their own mental health concerns within the past six months, or who had participated in any research study related to mental health interventions within the past year.

### 2.2 Method

#### 2.2.1 Determining the sample size

According to Kendall’s method for calculating sample size (17), the sample content is 5~10 times the number of independent variables in the study. In this study, the independent variables are the demographic data of children with ASD and their caregivers, requiring consideration of 17 variables. Considering that a 20% rate of invalid questionnaires or loss of follow-up may occur during the research process, the final sample size was determined to be 102~204 cases. Based on previous studies indicating that 68.80% of caregivers of children with ASD experience psychological disorders (18), the sample size was calculated using an incidence-based sample calculation formula: n= (z_(α⁄2)×pq)/δ^2 , p is the present-hazard rate, q=1-p, δ is the tolerance error, generally taken as 0.1*p, z_(α⁄2) is the significance test statistic, *zα*/2 = 1.96 when α=0.05 (19). After accounting for an estimated 20% dropout rate during the study period, the required sample size was determined to be 219 participants. A total of 278 caregivers participated in the survey, of which 245 met the inclusion criteria. Thirty-three caregivers were excluded for the following reasons: not meeting the inclusion criteria (n=25) and refusal to participate (n=8). Among the 245 eligible caregivers who agreed to participate, an additional 32 individuals were excluded due to incomplete questionnaires (n=20) or logically contradictory responses (n=12), resulting in a final sample size of 213 participants with a response rate of 86.9%. All participants in this study were informed and consented beforehand.

The coding and assignment of each variable are detailed in **Supplementary Table 1**.

#### 2.2.2 Survey tools

Demographic information was collected using a self-designed questionnaire covering child characteristics (e.g., gender, age, illness severity, payment method, comorbidities, treatment duration, only-child status) and caregiver characteristics (e.g., gender, age, education level, place of origin, employment status, marital status, monthly family income per capita, daily care duration, history of chronic diseases, and knowledge of ASD). The severity of ASD in children was determined by the treating clinicians based on the DSM-5 criteria, using clinical judgment of the level of support required. Severity was categorized as mild, moderate, or severe as documented in the children’s medical records. Comorbidities referred to co-occurring neurodevelopmental or medical conditions in the child with ASD, including but not limited to attention-deficit/hyperactivity disorder (ADHD), intellectual disability, epilepsy, anxiety disorders, and gastrointestinal disorders, as reported by the caregivers and confirmed by medical records.

To ensure the reliability and validity of this instrument in our sample, we assessed its psychometric properties. The internal consistency for the multi-item sections of the questionnaire was acceptable, with a Cronbach’s α of 0.79. An exploratory factor analysis revealed a Kaiser-Meyer-Olkin (KMO) measure of 0.81, and Bartlett’s test of sphericity was significant (p<0.001), confirming the suitability for factor analysis. The analysis extracted five factors with eigenvalues greater than 1, accounting for 68.5% of the total variance.

Psychological distress was assessed using the Symptom Checklist-90 (SCL-90), which was introduced in China in 1984 and is widely used to measure clinical psychiatric symptoms and mental health status. The scale consists of 90 items covering 10 symptom dimensions: somatization, obsessive-compulsive symptoms, interpersonal sensitivity, depression, anxiety, hostility, phobic anxiety, paranoid ideation, psychoticism, and sleep and appetite disturbances. The total score ranges from 90 to 450 based on a 5-point Likert scale (1 = none; 2 = mild; 3 = moderate; 4 = severe; 5 = extremely severe). A total score of ≥ 160 was defined as indicating psychological distress, while a score < 160 was considered to reflect mental well-being (20).

The use of the SCL-90 total score cutoff of 160 to define a dichotomous outcome was based on the following considerations: first, this cutoff was established in Chinese normative studies and has been widely adopted in epidemiological and clinical research within China to screen for individuals with a clinically significant symptom burden (20–22); second, a dichotomous outcome is operationally clear and facilitates the application and interpretation of the machine learning classification algorithms (LASSO and RF) employed in this study. The Cronbach’s α coefficients for the SCL-90 items range from 0.77 to 0.99, and in the present study, they ranged from 0.87 to 0.95.

#### 2.2.3 Statistical analysis

##### 2.2.3.1 LASSO regression

LASSO regression was employed for variable selection and to mitigate overfitting in high-dimensional data. This technique constructs a penalty function to shrink the coefficients of less important variables toward zero. The optimal regularization parameter (λ) was determined through 10-fold cross-validation with 2000 iterations. The value of λ that yielded the minimum mean cross-validated error (λmin) was selected to fit the final model, achieving a balance between model complexity and predictive accuracy (23, 24). The analysis was performed using the glmnet package (version 4.1-8) in R 4.4.2 software.

##### 2.2.3.2 RF

The RF is a non-parametric ensemble machine learning method that constructs multiple decision trees and aggregates their results to improve predictive accuracy and avoid overfitting. The dataset was randomly divided into a training set (150) and a validation set (63) using stratified sampling at a 7:3 ratio to maintain similarity in the distribution of results across the two subsets. Model optimization was achieved by tuning two key parameters: mtry (the number of variables randomly sampled as candidates at each split) and ntree (the number of trees in the forest). A grid search was performed over a range of mtry values (from 1 to the total number of predictors), and the model’s performance was monitored using the Out-of-Bag (OOB) error estimate (25–27). The final model selected the parameter combination that minimized the out-of-bag (OOB) error through ten-fold cross-validation, with corresponding settings of mtry = 2 and ntree = 1000 (at which point the error had stabilized). This model was implemented using the ‘randomForest’ package (version 4.7-1.1) in R.

##### 2.2.3.3 Model evaluation

The efficacy of the model was evaluated using four indicators: sensitivity, specificity, accuracy, F1 value and the area under the ROC curve (AUC). Among them, the AUC value is used to measure the truthfulness of the model’s prediction. When the AUC is greater than 0.5, the model has practical application value; the closer the AUC is to 1.0, the higher the truthfulness of the prediction model (28).

##### 2.2.3.4 Decision curve analysis

Decision Curve Analysis (DCA) (29, 30) is a mathematical model used to evaluate the usability and benefits of predictive tools, quantitatively describing the clinical utility of a model by analyzing the relationship between threshold probability and net benefit. If it is assumed that there exists a probability \(P_t \), and clinical action is taken when the positive probability is greater than \(P_t \), then \(P_t \) is referred to as the threshold probability. The general option is to select the incidence rate of the disease as the threshold probability. Net benefit (NB) refers to the proportion of net positives in the entire sample at a certain threshold probability. The formula is: NB=(TP−odds(T)*P_t/(1−P_t ))/N, Using the “rmda” package in R 4.4.2 statistical analysis software, decision curves were drawn with net return rate as the vertical axis and threshold probability as the horizontal axis.

##### 2.2.3.5 Chi-square test

A chi-square test was employed to conduct descriptive analyzes of the demographic characteristics of caregivers of preschool-aged children with ASD, and to analyze the distribution of subscale scores on the SCL-90. All statistical analyzes in this study were performed using R software version 4.4.2.

#### 2.2.4 Quality control

Strict adherence to the inclusion and exclusion criteria for the selection of research subjects, conducting one-on-one questionnaire surveys of the research subjects. Researchers must ensure the completeness of the questionnaire when distributing it, and if any parts are found to be missing, they should be corrected in a timely manner. Data organization is initially carried out by the researcher. If the answers show obvious regularities, and the majority of the options are consistent, they are considered invalid. Invalid questionnaires are excluded, and a dedicated database is established to ensure the accuracy and reliability of the data. Suspicious data is re-checked against the original questionnaire.

To address potential overfitting due to the sample size and model complexity, we implemented the following measures. First, internal validation with Bootstrap correction: for the Random Forest model, we performed 1000 Bootstrap iterations on the training set. The reported performance metric (AUC) is the Bootstrap-corrected estimate, providing a more conservative and realistic assessment of model performance. Second, we constructed a traditional logistic regression model using the same predictors selected by LASSO. This model serves as a simpler, more interpretable benchmark to contextualize the performance of the complex Random Forest model. Finally, AUC values are reported with their 95% confidence intervals to quantify estimation uncertainty.

## 3 Results

### 3.1 Descriptive statistics of preschool children with ASD and their caregivers

Based on the SCL-90 total score cutoff (≥160) described in the Methods section, participants were classified into two groups: the high psychological distress group (n=105) and the low psychological distress group (n=108). This study included a total of 213 subjects, among which there were 105 in the high psychological distress group and 108 in the low psychological distress group. The differences in the composition ratios of children’s age, degree of illness, medical payment method, comorbidities, treatment duration, work status, marital status, monthly family income, daily care duration, presence or absence of chronic diseases, and knowledge level about ASD between the group with psychological distress and the group with mental health had statistical significance (P<0.05). Analysis of differences in demographic characteristics is presented in **Table 1** and **Supplementary Table 2**.

**Table 1:** Demographic differences in psychological distress among caregivers of preschool children with ASD.

Characteristic	High psychological distress	Low psychological distress	χ^2^	*P* value
	105	108		
Age of the child (%)			11.545	0.003
2–3 years old	17 (16.2)	33 (30.6)		
4–5 years old	49 (46.7)	55 (50.9)		
6–7 years old	39 (37.1)	20 (18.5)		
Degree of illness (%)			20.278	<0.001
Mild	33 (31.4)	66 (61.1)		
Moderate	45 (42.9)	31 (28.7)		
Severe	27 (25.7)	11 (10.2)		
Medical payment method (%)			11.735	0.008
At his own expense	29 (27.6)	11 (10.2)		
Medical insurance	46 (43.8)	52 (48.1)		
Disabled Persons' Federation subsidy	16 (15.2)	21 (19.4)		
All of the above	14 (13.3)	24 (22.2)		
Complications (%)			36.505	<0.001
Yes	50 (47.6)	11 (10.2)		
No	55 (52.4)	97 (89.8)		
Treatment time (%)			9.731	0.021
0–4 months	30 (28.6)	44 (40.7)		
5–8 months	21 (20.0)	26 (24.1)		
9–12 months	16 (15.2)	19 (17.6)		
More than 12 months	38 (36.2)	19 (17.6)		
Working conditions (%)			24.315	<0.001
Incumbency	51 (48.6)	84 (77.8)		
Unemployed	48 (45.7)	16 (14.8)		
Retirement	6 (5.7)	8 (7.4)		
Marital status (%)			22.368	<0.001
Unmarried	4 (3.8)	2 (1.9)		
Married	76 (72.4)	103 (95.4)		
Divorced/separated	22 (21.0)	2 (1.9)		
Widowed	3 (2.9)	1 (0.9)		
Per capita monthly household income (RMB) (%)			9.454	0.024
≤3000	28 (26.7)	15 (13.9)		
3001-5000	26 (24.8)	34 (31.5)		
5001-7000	26 (24.8)	41 (38.0)		
>7000	25 (23.8)	18 (16.7)		
Length of daily care (h) (%)			21.026	<0.001
≤3h	8 (7.6)	29 (26.9)		
4-6h	12 (11.4)	19 (17.6)		
7-9h	9 (8.6)	13 (12.0)		
>10h	76 (72.4)	47 (43.5)		
Do you have any chronic diseases? (%)			14.631	<0.001
Yes	20 (19.0)	3 (2.8)		
No	85 (81.0)	105 (97.2)		
Knowledge about ASD (%)			12.842	0.005
Not at all	15 (14.3)	3 (2.8)		
A little bit	49 (46.7)	44 (40.7)		
Basic understanding	30 (28.6)	49 (45.4)		
Completely understand	11 (10.5)	12 (11.1)		

#### 3.1.1 SCL-90 subscale analysis

To further determine whether the level of psychological distress observed in our caregiver sample exceeded that of the general population, we compared the SCL-90 scores of the 213 caregivers with the Chinese national norms (20). As shown in **Table 2**, the total SCL-90 score of the caregiver group (141.96 ± 58.03) was significantly higher than that of the national norms (129.96 ± 38.76, P < 0.001). Similarly, caregivers scored significantly higher on most subscales, including somatization, obsessive-compulsive symptoms, depression, anxiety, hostility, phobic anxiety, psychoticism, and sleep/appetite disturbances (all P < 0.001). In contrast, interpersonal sensitivity was slightly lower (P = 0.040), and paranoid ideation showed no significant difference (P = 0.742). These results indicate that family caregivers of preschool children with ASD experience a significantly higher overall burden of psychological distress compared to the general population.

**Table 2:** Comparison of SCL-90 scores between family caregivers of children with ASD and Chinese national norms.

Item	Caregiver group (N = 213)	National norms (N = 1388)	T-value	P-value
Total SCL-90 score	141.96 ± 58.03	129.96 ± 38.76	4.23	<0.001
Somatization	1.55 ± 0.64	1.37 ± 0.48	5.7	<0.001
Obsessive-compulsive	1.76 ± 0.76	1.62 ± 0.58	3.73	<0.001
Interpersonal sensitivity	1.58 ± 0.68	1.65 ± 0.61	-2.05	0.040
Depression	1.67 ± 0.75	1.50 ± 0.59	4.57	<0.001
Anxiety	1.58 ± 0.70	1.39 ± 0.43	5.58	<0.001
Hostility	1.60 ± 0.68	1.48 ± 0.56	3.55	<0.001
Phobic anxiety	1.41 ± 0.61	1.23 ± 0.41	6.03	<0.001
Paranoid ideation	1.44 ± 0.60	1.43 ± 0.57	0.33	0.742
Psychoticism	1.44 ± 0.61	1.29 ± 0.42	5.02	<0.001
Sleep and appetite disturbances	1.59 ± 0.71	—	—	—

Subscale analysis of the SCL-90 revealed consistent symptom patterns across the training set, validation set, and total dataset. In the training set, the most severe symptoms were obsessive-compulsive (1.758 ± 0.74), depression (1.734 ± 0.78), and sleep and eating disorders (1.695 ± 0.72). The validation set (obsession: 1.756 ± 0.86, depression: 1.639 ± 0.77, sleep and eating: 1.602 ± 0.74) and total dataset (obsession: 1.757 ± 0.78, depression: 1.706 ± 0.78, sleep and eating: 1.662 ± 0.73) exhibited identical patterns, with complete results detailed in **Supplementary Table 3**. Given the consistency across datasets, subsequent analyzes focused on the training set. All subscale distributions in the training set showed positive skewness, indicating that most individuals had low symptom scores approaching ‘healthy’ levels. This skewness pattern is consistent with what is typically observed in general population surveys, where the majority report minimal symptoms while a minority exhibit elevated scores. Nevertheless, as demonstrated in **Table 2**, the absolute SCL-90 scores of our caregiver sample were significantly higher than national norms, indicating an elevated burden of psychological distress in this population. The three symptoms with highest prevalence were obsessive-compulsive behaviors, depression, and sleep/eating disturbances, with comprehensive results presented in **Supplementary Figure 1**.

### 3.2 LASSO regression analysis

**Figure 1:**
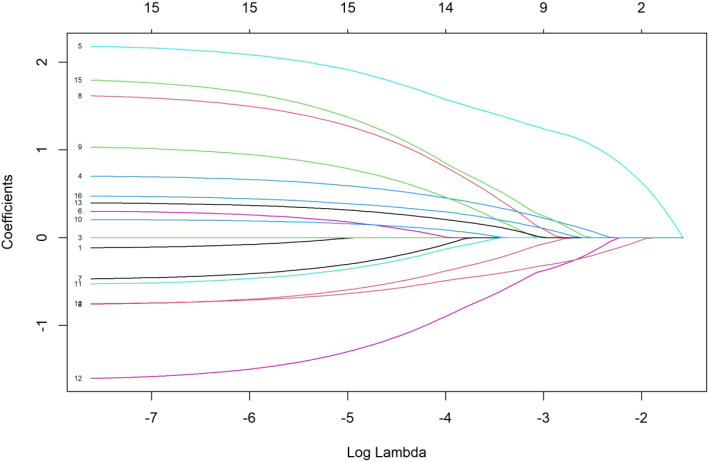
Trajectory of regression coefficients in the LASSO model. 1 for child's gender, 2 for child's age, 3 for severity of illness, 4 for medical payment method, 5 for comorbidities, 6 for treatment duration, 7 for only child, 8 for caregiver's gender, 9 for caregiver's age, 10 for education level, 11 for origin of students, 12 for work status, 13 for marital status, 14 for monthly family income, 15 for daily care duration, 16 for own chronic diseases, 17 for ASD knowledge level.

**Figure 2:**
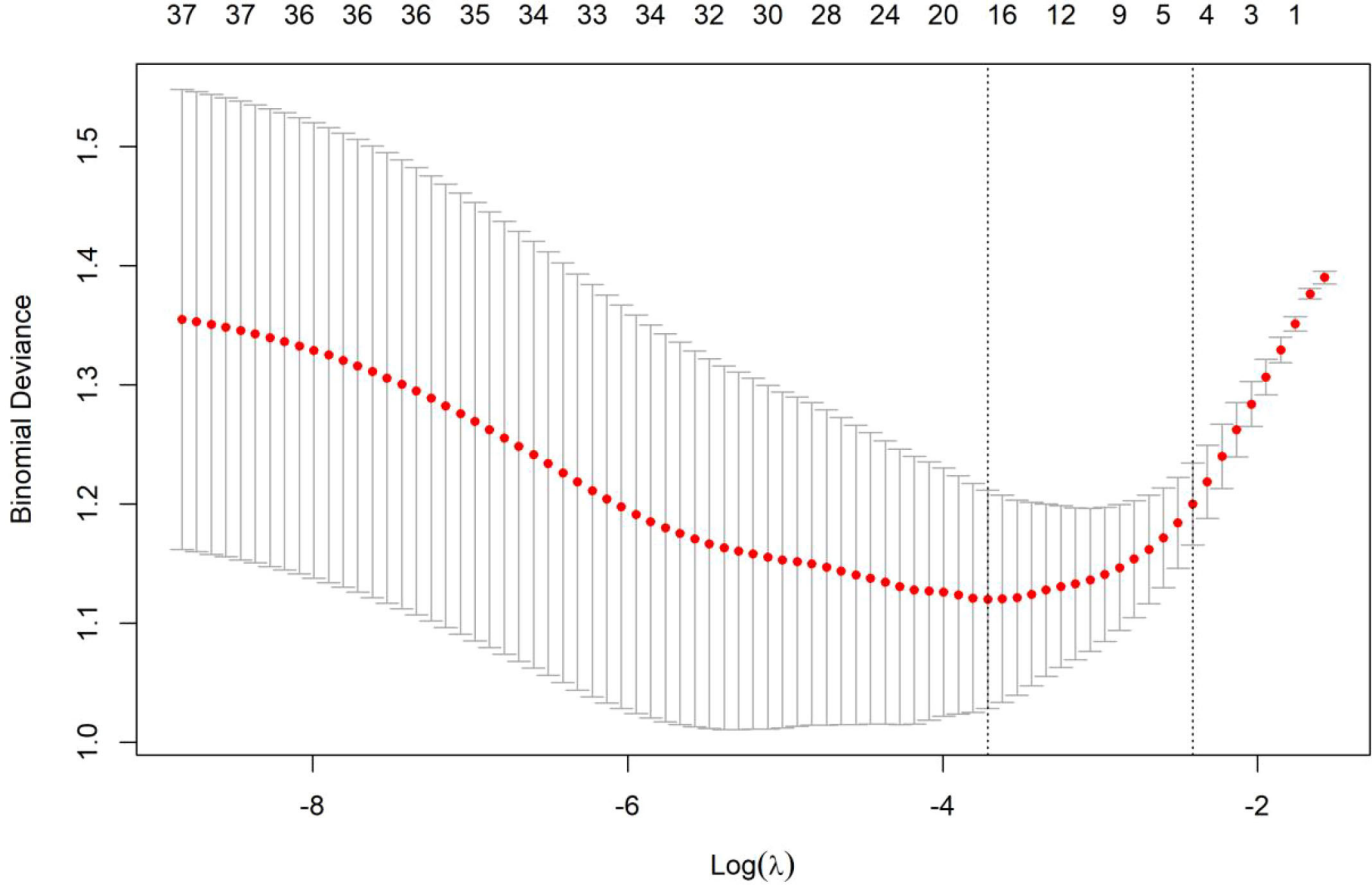
The variation process of the optimal penalty coefficient λ in the LASSO regression model.

Seventeen demographic characteristics of the factors related to psychological distress were included in the LASSO regression model. Using 10-fold cross-validation, the critical parameter value of the model (λ= 0.0267) was determined. The variable screening process is shown in **Figures 1**, **2**. Secondly, the coefficients of the factors that were not significantly associated with mental disorders were shrunken to 0 and excluded. Twelve variables were finally selected, namely child age, medical payment method, comorbidity, caregiver gender, caregiver age, education, working condition, marital status, family monthly income, daily care duration, own chronic diseases, ASD knowledge, etc., and the regression coefficients are shown in **Table 3**.

**Table 3:** variables and their regression coefficients.

Variables	Regression coefficient	Variables	Regression coefficient
Age of the child	-0.279	Working conditions	-0.045
Medical payment method	0.379	Marital status	-0.706
Complications	1.451	Per capita monthly household income	0.150
Caregiver gender	0.559	Length of daily care	-0.433
Age of caregiver	0.284	Do you have any chronic diseases?	0.631
Education level	0.037	Knowledge about ASD	0.234

### 3.3 Random forest algorithm

#### 3.3.1 Variable importance ranking

**Figure 3:**
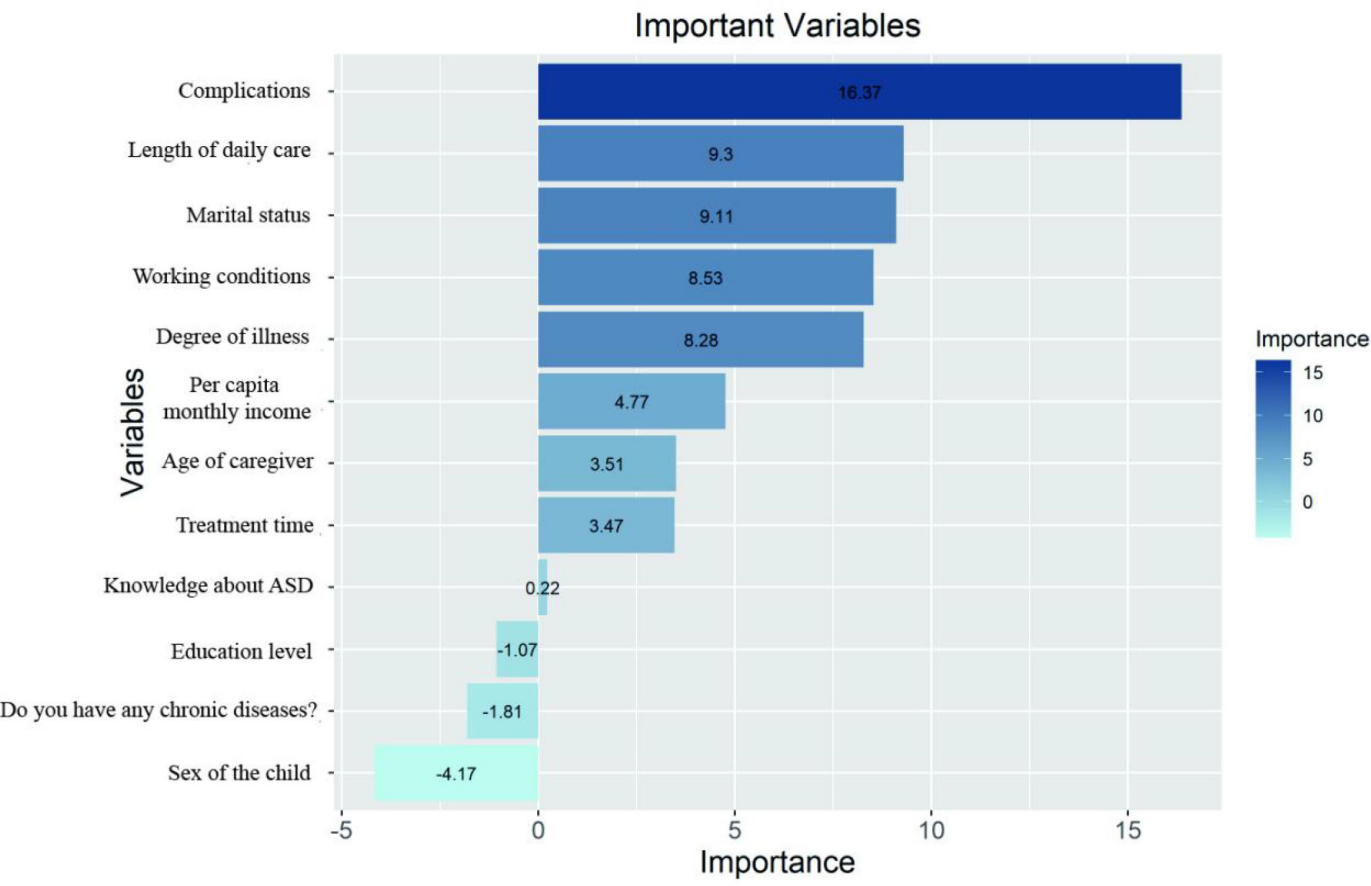
Ranks the importance of variables in the RF for caregiver psychological distress risk factors.

The variables screened by LASSO regression were included in the random forest model established on the validation set. **Figure 3** shows the feature importance ranking, demonstrating the contribution of various influencing factors to the prediction model of caregivers’ psychological distress. The top 7 influencing factors are, in order: work status, marital status, comorbidities, disease severity, monthly family income, daily care duration, and treatment duration.

#### 3.3.2 Model building and evaluation

**Figure 4:**
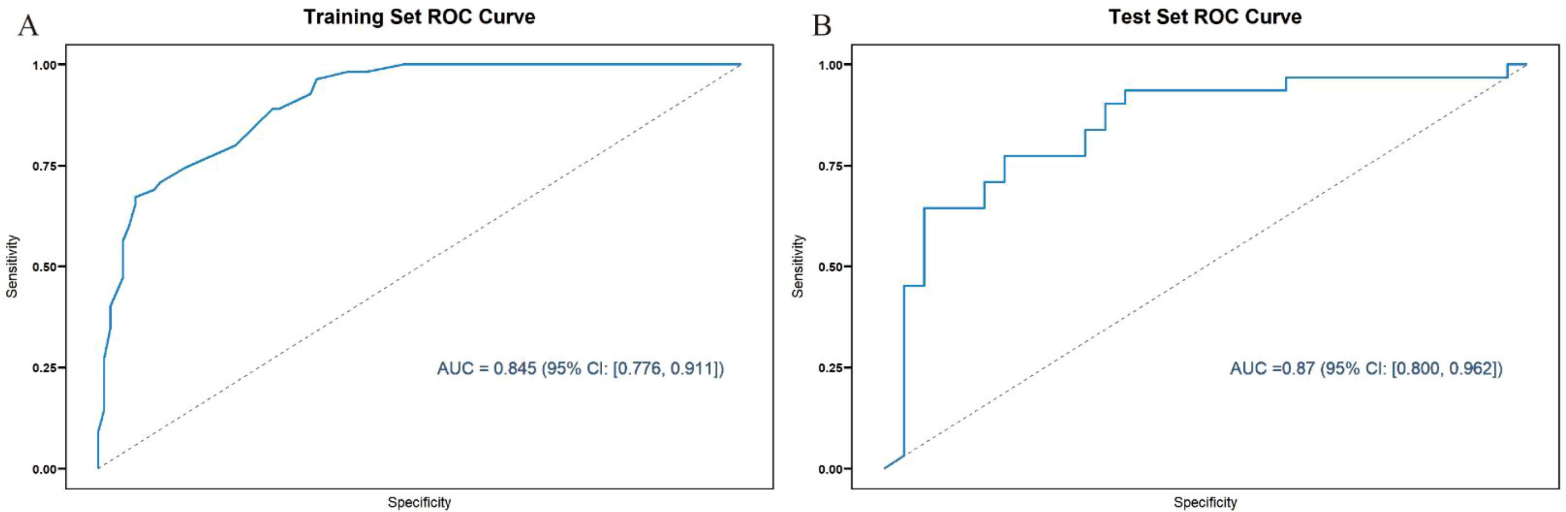
ROC curves of the Random Forest model. **(A)** Training set, AUC = 0.845 (95% CI: 0.776–0.911). **(B)** Test set, AUC = 0.87 (95% CI: 0.800–0.962).

LASSO regression identified several significant predictors of psychological distress among caregivers of preschool-aged children with ASD, including employment status, marital status, comorbidities, disease severity, monthly household income, daily caregiving duration, treatment duration, understanding of ASD, educational level, age, presence of chronic diseases, and the child’s gender. An RF model was established using the training set, yielding a corrected AUC of 0.845 [95% CI: (0.776, 0.911)] (**Figure 4A**). Performance metrics for the training set model indicated an accuracy of 0.933, sensitivity of 0.893, specificity of 0.913, F1 score of 0.901, and the confusion matrix is shown in **Supplementary Figure 2**. When evaluated on the test set, the bootstrap-corrected AUC was 0.87 [95% CI: (0.800, 0.962)] (**Figure 4B**), demonstrating reasonable generalization capability. Performance metrics for the validation set included an accuracy of 0.710, sensitivity of 0.733, specificity of 0.727, and F1 score of 0.721, with the confusion matrix detailed in **Supplementary Figure 3**. The high specificity (0.913) indicates that the model effectively and accurately identifies caregivers without psychological distress.

#### 3.3.3 Logistic regression

The robustness of the results was validated using logistic regression analysis. The model performance metrics obtained through ten-fold cross-validation on the training set demonstrated: accuracy of 0.84, sensitivity of 0.881, specificity of 0.797, F1 score of 0.848, and AUC of 0.894 [95% CI: (0.800, 0.962)]. Performance metrics on the test set showed: accuracy of 0.652, sensitivity of 0.625, specificity of 0.86, F1 score of 0.761, and AUC of 0.767 [95% CI: (0.650, 0.884)], as detailed in the **Supplementary Figure 4**.

As summarized in **Table 4**, the RF model outperformed logistic regression on most metrics in both training and test sets.

**Table 4:** Performance metrics of RF and logistic regression models on training and test sets.

Model	Dataset	AUC (95% CI)	Accuracy	Sensitivity	Specificity	F1 score
RF	Training	0.845 [0.776–0.911]	0.933	0.893	0.913	0.901
	Test	0.870 [0.800–0.962]	0.71	0.733	0.727	0.721
Logistic Regression	Training	0.894 [0.800–0.962]	0.840	0.881	0.797	0.848
	Test	0.767 [0.650–0.884]	0.652	0.625	0.860	0.761

#### 3.3.4 Decision curve analysis

**Figure 5:**
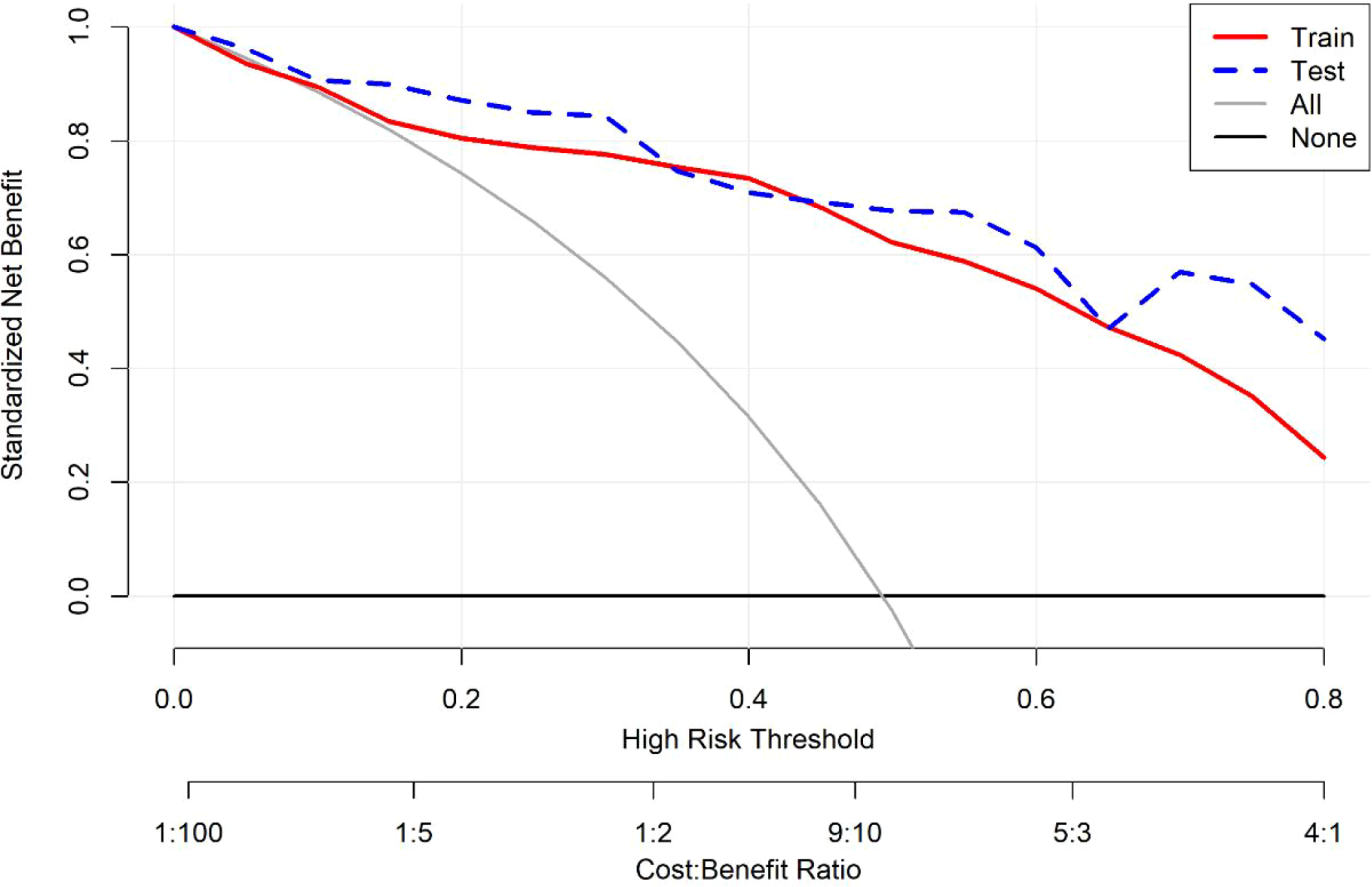
Decision curve predicted by the RF model.

DCA results show that within the threshold probability range of 0 to 0.85, the decision curves of the random forest prediction model are above both the full-positive line and the full-negative line, suggesting potential clinical net benefit (**Figure 5**). When setting the probability threshold at 0.5, the net benefit of the training set was 44.43%, and that of the test set was 27.76%. It should be noted that these findings are based on internal validation; external validation in independent samples is necessary to confirm the model’s clinical utility.

## 4 Discussion

This study applied the RF algorithm combined with LASSO regression analysis to identify risk factors for psychological distress among family caregivers of preschool-aged children with ASD. The results indicated that factors including the child’s age, medical payment method, comorbidities, caregiver’s gender, age, educational level, employment status, marital status, monthly household income, daily care duration, chronic disease status, and understanding of ASD all had significant impacts. The RF model achieved an AUC value of 0.845 in the training set and 0.87 in the test set. Relevant studies have demonstrated the substantial potential of machine learning in early prediction of psychological distress onset (31), while subsequent decision curve analysis revealed that this model exhibited favorable net benefit rates and clinical applicability (32). Through RF feature importance ranking, this study identified five most critical influencing factors: comorbidities, daily care duration, marital status, severity of the condition, and employment status (importance scores >8.0). These findings align with previous research regarding the effects of ASD symptom severity, insufficient social support, economic pressure, and comorbidity status on caregiver mental health (33–35).

Our finding that family caregivers of preschool children with ASD had significantly higher SCL-90 total scores and higher scores on most subscales compared to Chinese national norms is consistent with previous research documenting elevated psychological distress in ASD caregiver populations (33, 34). Analysis of the SCL-90 scale and its subscales revealed the multidimensional nature of psychological distress. Obsessive-compulsive, depressive, and sleep/appetite symptoms scored higher in most family caregivers. Previous research suggests that caregivers with higher obsessive-compulsive symptoms may express psychological distress through alternative, non-somatic pathways, where initial somatic and phobic anxiety symptoms are often replaced by compulsive behaviors as the care recipient’s condition progresses. High obsessive-compulsive scores may be associated with the relentless, routine-driven demands of ASD caregiving and intrusive worries about the child’s future (36, 37). The chronic stress and exhaustion of high-intensity caregiving may contribute to the development and exacerbation of depressive symptoms and sleep disturbances (38, 39). This indicates that mental health interventions for caregivers should be symptom-informed—such as incorporating cognitive-behavioral techniques to manage repetitive worries, behavioral activation for depression, and sleep hygiene education—rather than offering only generic stress management programs.

The prominence of comorbidities in our feature importance analysis warrants deeper theoretical consideration. Children with ASD frequently present with co-occurring conditions such as ADHD, intellectual disabilities, and anxiety disorders, which exponentially increase caregiving demands. The mechanism underlying this relationship involves the cumulative burden hypothesis: each additional condition introduces unique behavioral challenges, therapeutic requirements, and monitoring needs that strain caregiver resources (40). Children with both autism and visual impairment exemplify the compounded challenges faced by families. Traditional autism intervention models, which heavily rely on visual input, are less effective for children with visual impairments and require specialized adaptations (41). This imposes additional demands on support services, further exacerbating caregiver burden. Moreover, support services are often fragmented across different clinics—each focusing separately on autism, visual impairment, or intellectual disability—with little attention to their co-occurrence. As previous research has highlighted, families often struggle to navigate this disjointed support system, leaving parents forced to act as “spiders in the web,” coordinating between various professionals and services (42).This caregiving burden compounds with direct care needs, creating multidimensional stress for caregivers. The mechanisms of comorbidity effects involve not only the accumulation of clinical burdens but also systemic difficulties in accessing appropriate and coordinated care. Each additional condition introduces unique behavioral challenges, therapeutic requirements, and service coordination demands, which grow exponentially and deplete caregiver resources (43).The high ranking of daily care duration reflects the intensive, often lifelong nature of ASD caregiving. Unlike other developmental disorders, ASD presents unique challenges including communication deficits, social skills deficits, and behavioral dysregulation that require continuous intervention. This aligns with autism-specific care models emphasizing the need for structured routines, social skills training, and behavior management strategies that demand substantial time investment from caregivers (44).Children with autism, dependent on parental care and needs, require their parents to make arrangements for their own lives, including work life. Research has revealed that parents faced occupational barriers and were unable to take every job, had difficulty remaining employed, and worked fewer hours due to caregiving demands for their children, therefore, had lower incomes (45–47); and parents of children with special needs, such as parents of children with ASD (5), experienced greater work-family conflict and needed more workplace flexibility than parents of typically developing children which might result in consequences for their physical and mental health (48).Mutual support among family members can serve as a mediating factor for individuals in coping with psychological distress. Understanding and communication among family members of children with illnesses can alleviate the burden of care for each other (49). This can reduce the risk of the occurrence of psychological distress (50). Children with severe ASD often exhibit repetitive behaviors, meltdowns, communication challenges, and other issues that require constant attention and management from caregivers. For instance, a child may repeatedly ask the same question or engage in the same behavior, requiring caregivers to respond and redirect patiently. This constant high demand leads to physical and mental exhaustion for caregivers, which in turn results in ineffective coping strategies and increased parental stress. When compared to studies of caregivers for children with other developmental disorders, our findings highlight several ASD-specific considerations. The prominence of daily care duration and comorbidities distinguishes ASD caregiver burden from that associated with conditions like cerebral palsy or Down syndrome, where physical care needs may be more prominent but behavioral management demands are less intensive. This underscores the need for disorder-specific intervention approaches rather than generic caregiver support programs (51). The negative correlation coefficient associated with daily caregiving duration may be explained by a specific psychological dynamic observed during prolonged intervention for ASD. When a child’s progress fails to meet the initial expectations set by family caregivers—such as slow improvement in social skills, language expression, or reduction in stereotyped behaviors—caregivers are prone to experience intense frustration. They may begin to doubt the effectiveness of the chosen intervention or question the value of their considerable investment of time and effort. This accumulation of negative emotions can significantly exacerbate psychological distress (52).

Based on the factors identified in this study, prioritizing interventions through feature importance ranking provides a potential stratified basis for intervention priorities. First, managing complications should be the primary focus, advocating for person-centered integrated care. A four-party collaborative mechanism involving home, school, community, and hospital should be established to simultaneously address autism spectrum disorder and comorbid conditions, thereby mitigating complex behavioral challenges (53). Second, marital status and employment status highlight the critical role of socioeconomic support systems. Interventions such as caregiver partner counseling or workplace flexibility policies are recommended, alongside stress diaries and nonviolent communication to improve family interactions. Finally, while daily care duration is significant, its adjustability is limited. Efforts should focus on respite care services to alleviate caregiving intensity, offering temporary foster care, in-home care, and caregiver training (54).

In this study, we constructed both RF and logistic regression models to predict psychological distress among caregivers of children with ASD. On internal validation, the RF model demonstrated acceptable discrimination (AUC = 0.87) and outperformed logistic regression on most metrics. However, these results should be interpreted as preliminary, as true generalizability can only be established through external validation in multi-center cohorts. This suggests that the ensemble learning approach of random forest can better capture the complex, potentially nonlinear relationships between risk factors and psychological distress within this population. The algorithm’s ability to handle interactions between variables and its robustness against outliers may contribute to its enhanced performance. The consistency in key risk factors identified by both methods—including comorbidity status, daily care duration, marital status, disease severity, and employment status—provides convergent validity for these determinants, while the superior performance of random forest indicates that machine learning approaches may offer advantages over traditional statistical methods in predicting complex psychosocial outcomes for caregivers of children with autism.

Based on our findings, we propose several evidence-based interventions. (1) Workplace accommodations: Implement flexible work arrangements, telecommuting options, and adjusted schedules for caregivers of children with ASD. Employers should develop formal caregiver support policies recognizing the unique challenges faced by this population. (2) Temporary respite services: Establish government-subsidized temporary childcare services specifically trained in ASD management to provide caregivers with essential breaks from continuous care demands. (3) Targeted mental health screening: Develop routine screening programs for psychological disorders among caregivers of children with ASD, particularly focusing on those reporting extended daily care duration or managing multiple comorbidities. (4) Financial support mechanisms: Create sliding-scale subsidy programs for low-income families caring for children with ASD, addressing the economic burden identified through the monthly household income variable.

The limitations and shortcomings of this study are primarily reflected in the following aspects. First, the convenience sampling method employed to recruit participants from only three institutions in Urumqi may introduce selection bias, as caregivers who agreed to participate might differ systematically from those who declined or from the broader population of ASD caregivers. Consequently, the findings may not be effectively generalized to other cities or regions across China. Second, the sample size (N = 213) is modest relative to the number of predictors (17 variables). While we employed LASSO for variable selection and bootstrap correction to mitigate overfitting, the risk of overfitting cannot be entirely ruled out given the complexity of the machine learning models and the sample size. Third, as a cross-sectional study design was adopted, this research cannot thoroughly explore causal relationships, necessitating more in-depth investigations in subsequent studies. Fourth, the operationalization of psychological distress has inherent limitations. Although justified for our primary aim, dichotomizing the multidimensional SCL-90 into a single binary outcome inevitably oversimplifies the complex phenomenological experience of distress and loses information about specific symptom patterns. Future research could treat the SCL-90 subscales as multiple continuous outcomes in a multivariate modeling framework or employ more comprehensive diagnostic interviews to capture clinical disorders. Therefore, we strongly recommend external validation of this model using larger, multi-center, prospectively collected datasets before any clinical implementation is considered.

In summary, this study employed LASSO regression to select variables, ensuring that those included in the RF evaluation model are highly correlated with the psychological distress of preschool-aged ASD children’s family caregivers. By utilizing the importance ranking of the random forest, we discovered the ranking of contributing factors affecting caregiver psychological distress. The AUC value of the risk assessment model is above 0.8 in both the test and validation sets, demonstrating good predictive performance. It possesses commendable sensitivity, specificity, and accuracy, enabling the accurate identification of individuals at risk of psychological distress related to preschool-aged ASD children’s caregivers. This allows for personalized mental health intervention guidance and, to a certain extent, can prevent the risk of psychological distress in ASD children’s family caregivers, thereby promoting the therapeutic effects of ASD children.

## Data Availability

The raw data supporting the conclusions of this article will be made available by the authors, without undue reservation.
