# Supplementary material for: Factors associated with psychological distress among family caregivers of preschool children with autism: an analysis
Source: Front Psychiatry. 2026 May 20;17:1570322. doi: 10.3389/fpsyt.2026.1570322 (PMC13229856; doi:10.3389/fpsyt.2026.1570322)
Supplement: Supplementary file 1 [file DataSheet1.zip › Supplementary documents.docx]

**Supplementary Information**

Supplementary Table 1 Variable Encoding

| Characteristic | Code |
| --- | --- |
| Age of the child(%) | 1:2-3 years old |
|  | 2:4-5 years old |
|  | 3:6-7 years old |
| Degree of illness(%) | 1:Mild |
|  | 2:Moderate |
|  | 3:Severe |
| Medical payment method(%) | 1:At his own expense |
|  | 2:Medical insurance |
|  | 3:Disabled Persons' Federation subsidy |
|  | 4:All of the above |
| Complications(%) | 1:Yes |
|  | 2:No |
| Treatment time (%) | 1:0-4 months |
|  | 2:5-8 months |
|  | 3:9-12 months |
|  | 4:More than 12 months |
| Working conditions(%) | 1:Incumbency |
|  | 2:Unemployed |
|  | 3:Retirement |
| Marital status(%) | 1:Unmarried |
|  | 2:Married |
|  | 3:Divorced/separated |
|  | 4:Widowed |
| Per capita monthly household income(RMB)(%) | 1:≤3000 |
|  | 2:3001-5000 |
|  | 3:5001-7000 |
|  | 4:＞7000 |
| Length of daily care(h)(%) | 1:≤3h |
|  | 2:4-6h |
|  | 3:7-9h |
|  | 4:＞10h |
| Do you have any chronic diseases?(%) | 1:Yes |
|  | 2:No |
| Knowledge about ASD(%) | 1:Not at all |
|  | 2:A little bit |
|  | 3:Basic understanding |
|  | 4:Completely understand |
| Sex of the child(%) | 1:Male |
|  | 2:Female |
| Whether he is an only child(%) | 1:Yes |
|  | 2:No |
| Caregiver gender(%) | 1:Male |
|  | 2:Female |
| Age of caregiver (%) | 1:18-25 years old |
|  | 2:26-35 years old |
|  | 3:36-45 years old |
|  | 4:46-55 years old |
|  | 5:Over 56 years old |
| Education level(%) | 1:Primary school and below |
|  | 2:Junior middle school |
|  | 3:High School/Technical Secondary School |
|  | 4:College/Bachelor degree or above |
| Place of origin(%) | 1:Urumqi |
|  | 2:Other prefectures in Xinjiang |
|  | 3:Outside Xinjiang |
| SCL-90 | 1: ≥160 |
|  | 2:<160 |

Supplementary Table 2 Demographic Differences in Psychological Distress Among Caregivers of Preschool Children with ASD

| Characteristic | High psychological distress | Low psychological distress | χ^2^ | *P* value |
| --- | --- | --- | --- | --- |
|  | 105 | 108 |  |  |
| Sex of the child(%) |  |  | 0.079 | 0.779 |
| Male | 74(70.5) | 79(73.1) |  |  |
| Female | 31(29.5) | 29(26.9) |  |  |
| Whether he is an only child(%) |  |  | 0.119 | 0.73 |
| Yes | 55（52.4） | 53（49.1） |  |  |
| No | 50(47.6) | 55(50.9) |  |  |
| Caregiver gender(%) |  |  | 2.295 | 0.13 |
| Male | 24(22.9） | 15(13.9） |  |  |
| Female | 81(77.1) | 93(86.1) |  |  |
| Age of caregiver (%) |  |  | 6.929 | 0.14 |
| 18-25 years old | 6(5.7) | 2(1.9) |  |  |
| 26-35 years old | 56(53.3) | 54(50.0) |  |  |
| 36-45 years old | 26(24.8) | 39(36.1) |  |  |
| 46-55 years old | 5(4.8) | 7(6.5) |  |  |
| Over 56 years old | 12(11.4) | 6(5.6) |  |  |
| Education level(%) |  |  | 2.148 | 0.542 |
| Primary school and below | 6(5.7) | 8(7.4) |  |  |
| Junior middle school | 25(23.8) | 18(16.7) |  |  |
| High School/Technical Secondary School | 24(22.9) | 23(21.3) |  |  |
| College/Bachelor degree or above | 50(47.6) | 59(54.6) |  |  |
| Place of origin(%) |  |  | 0.585 | 0.746 |
| Urumqi | 26(24.8) | 27(25.0) |  |  |
| Other prefectures in Xinjiang | 67(63.8) | 72(66.7) |  |  |
| Outside Xinjiang | 12(11.4) | 9(8.3) |  |  |

Supplementary Table 3 Subscale Scores of SCL-90

| Subscale | Train set(Mean±SD) | Prevalence(%) | Test set(Mean±SD) | Prevalence(%) | Total data(Mean±SD) | Prevalence(%) |
| --- | --- | --- | --- | --- | --- | --- |
| obsessive_compulsive | 1.758±0.74 | 36.2 | 1.756±0.86 | 32.8 | 1.757±0.78 | 35.2 |
| depression | 1.734±0.78 | 32.2 | 1.639±0.77 | 28.1 | 1.706±0.78 | 31.0 |
| sleep_appetite | 1.695±0.72 | 30.9 | 1.602±0.74 | 28.1 | 1.662±0.73 | 28.6 |
| hostility | 1.636±0.70 | 26.2 | 1.585±0.76 | 23.4 | 1.626±0.71 | 26.8 |
| anxiety | 1.609±0.71 | 27.5 | 1.583±0.7 | 25.0 | 1.599±0.68 | 26.8 |
| interpersonal_sensitivity | 1.606±0.68 | 27.5 | 1.564±0.72 | 26.6 | 1.595±0.71 | 27.2 |
| somatization | 1.579±0.64 | 23.5 | 1.517±0.69 | 17.2 | 1.56±0.65 | 21.6 |
| psychoticism | 1.501±0.66 | 19.5 | 1.461±0.63 | 21.9 | 1.476±0.64 | 17.8 |
| paranoid_ideation | 1.477±0.63 | 20.1 | 1.417±0.62 | 14.1 | 1.472±0.63 | 20.7 |
| phobic_anxiety | 1.401±0.58 | 15.4 | 1.397±0.57 | 17.2 | 1.400±0.58 | 16.0 |

**
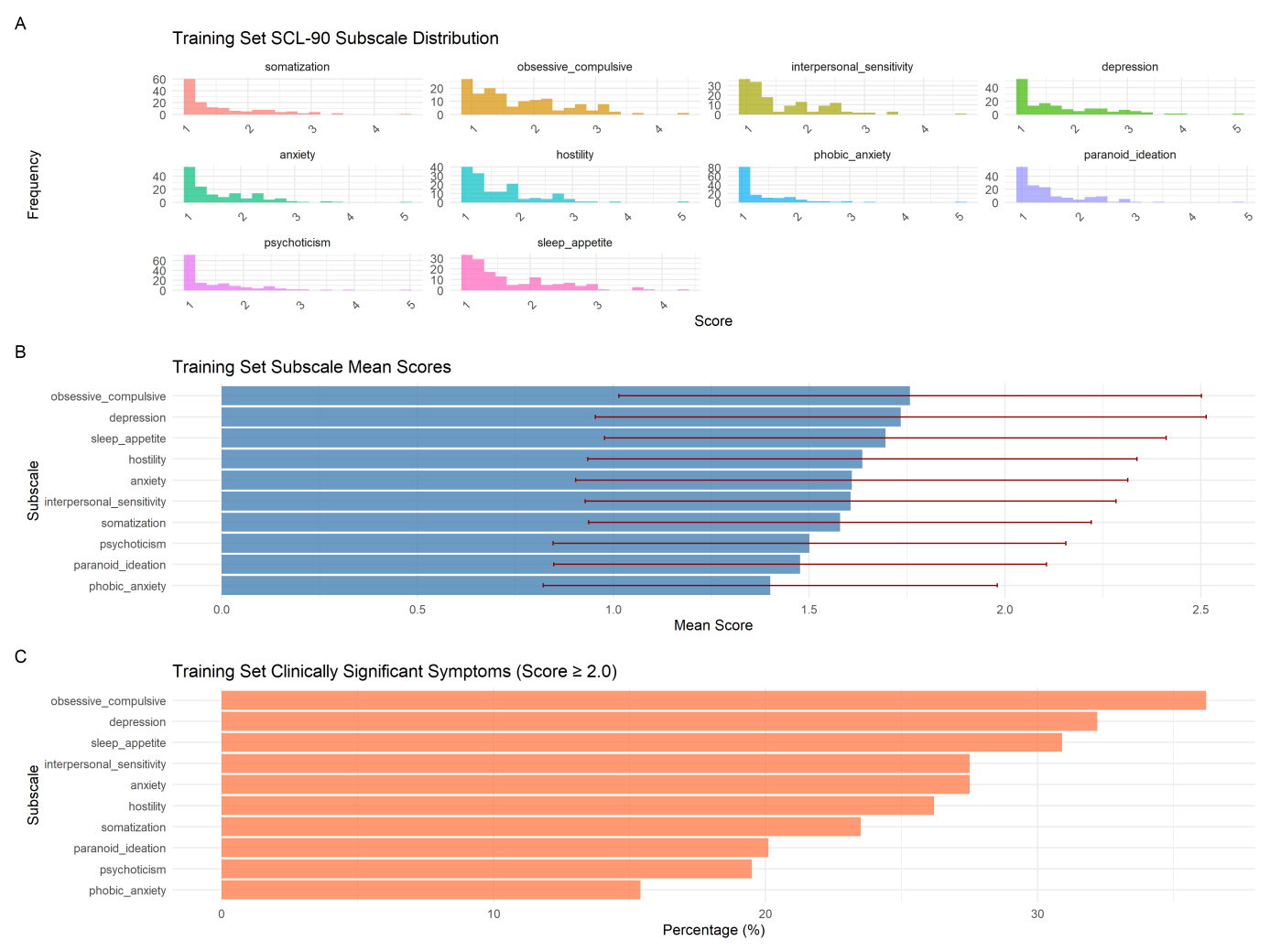
**

Supplementary Figure 1. Distribution of SCL-90 Subscale Scores in the Training Set


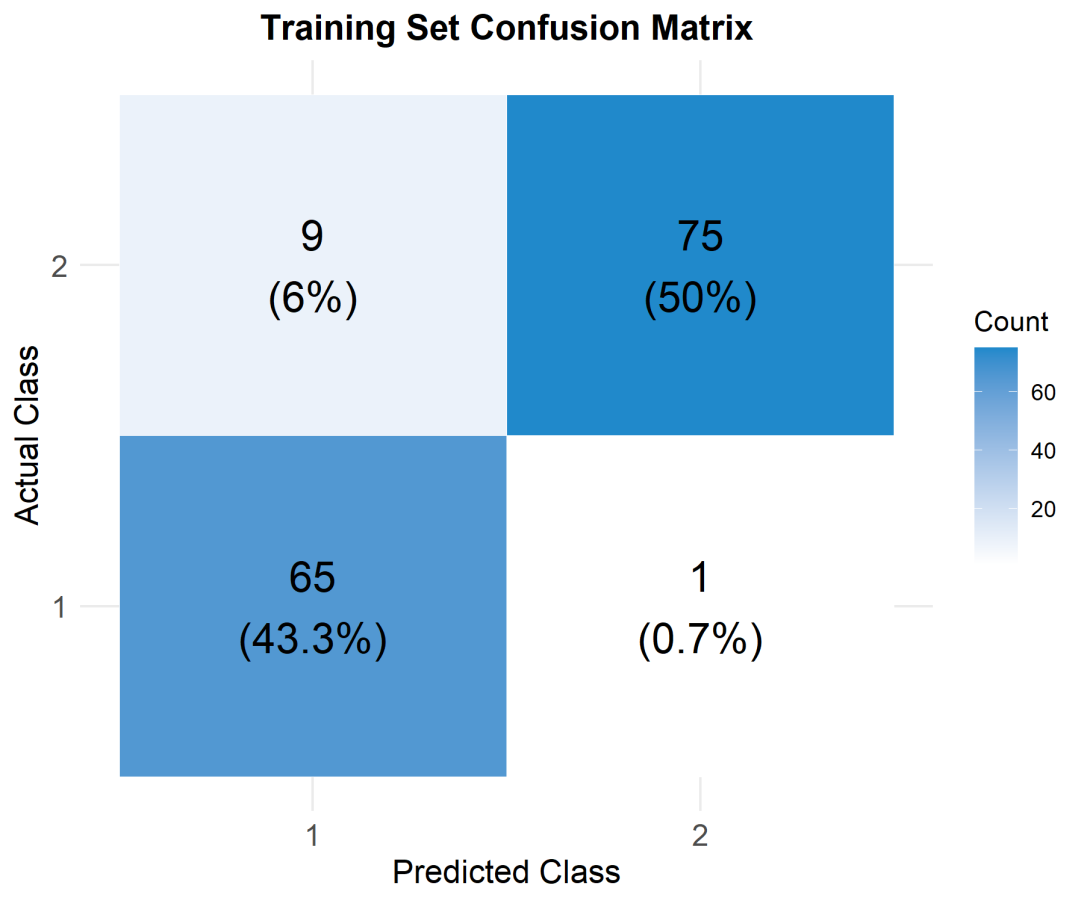


Supplementary Figure 2. Training Set Confusion Matrix


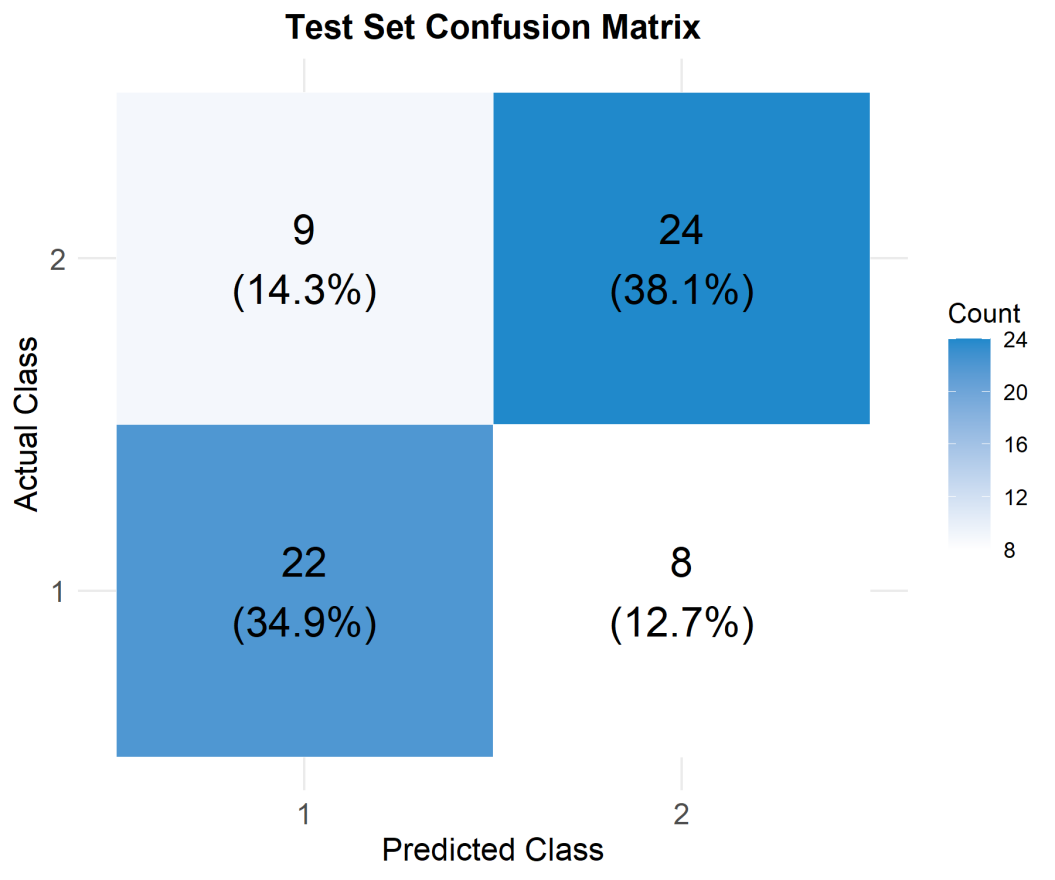


Supplementary Figure 3. Test Set Confusion Matrix


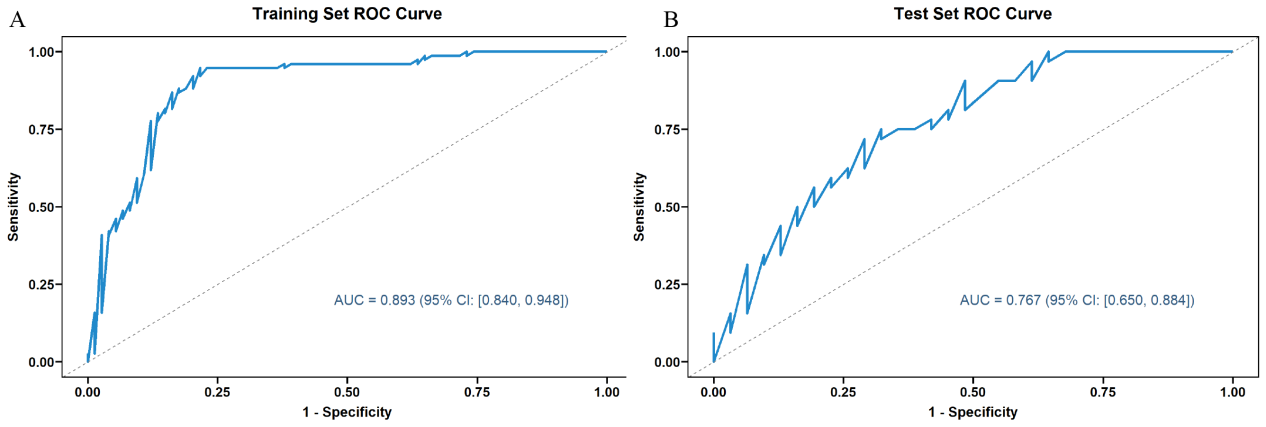


Supplementary Figure 4. Logistic Regression ROC Curve
